# Inhaled therapy guidance competency among respiratory nurses across hospital tiers in Eastern China: a cross-sectional study

**DOI:** 10.3389/fmed.2026.1817376

**Published:** 2026-04-20

**Authors:** Zhen-Yun Wu, Bo Jiang, Yan-Xia Han, Mei-e Niu, Qian Zhao

**Affiliations:** 1 Department of Respiratory Medicine, The First Affiliated Hospital of Soochow University, Suzhou, Jiangsu, China; 2 Yueyang Vocational Technical College, Yueyang, Hunan, China; 3 Department of Nursing, The First Affiliated Hospital of Soochow University, Suzhou, Jiangsu, China

**Keywords:** cross-sectional study, guidance competency, hospitals tiers, inhaled therapy, nurses

## Abstract

**Background:**

Inhaled therapy is critical for treating chronic airway diseases, yet the competency of respiratory nurses in providing guidance remains inconsistent. Few studies have explored the systemic competency disparities that are driven by a hierarchical distribution of healthcare resources. The aim of this study is to assess self-reported inhaled therapy guidance (ITG) competency among respiratory nurses across multiple-tier healthcare institutions, as well as to explore factors that affected such competency.

**Methods:**

A total of 962 respiratory nurses at multilevel hospitals in Jiangsu Province, Eastern China were investigated. We developed an ITG competency scale and evaluated its reliability and validity. Nurses rated themselves on a structured questionnaire that was designed to collect data on ITG competency in this population. The associated factors were determined using a descriptive statistical analysis, a correlation analysis, and a hierarchical multiple regression analysis. We followed the Strengthening the Reporting of Observational Studies in Epidemiology (STROBE) checklist for cross-sectional studies.

**Results:**

The ITG competency average score for respiratory nurses was (73.90 ± 9.42). Significant competency disparities were observed across all hospital tiers (*p* < 0.001), with the primary hospitals demonstrating higher rates of poor and lower proportions of good ratings than secondary/tertiary hospitals. For the knowledge dimension, tertiary hospitals had the fewest poor ratings, while primary hospitals exhibited the highest prevalence of poor ratings, although the proportion of good skill ratings remained comparable across all tiers (*p* > 0.05). Educational attainment, hospital grade, and training methodologies were associated with respiratory nurses’ competency at ITG.

**Conclusion:**

The respiratory nurses exhibited moderate levels of ITG competency, with a notable gap between their knowledge and skills. This gap was more pronounced in primary hospitals, suggesting an association with institutional resource contexts. These results highlight the need for training strategies tailored to each hospital tier, as well as enhanced resource support from tertiary centers to primary care. This would help promote more standardized training programs and reduce competency disparities across hospital tiers.

## 1 Introduction

Inhaled therapy is the method by which medications in aerosol or dry-powder form are delivered into the respiratory tract for local therapeutic effect via specialized devices (1). This is a critical method for treating patients with chronic airway diseases (2), and it offers advantages such as rapid onset, convenience, and minimal systemic adverse reactions. Various inhalation devices are used in clinical settings. These devices include pressurized metered-dose inhalers, dry-powder inhalers, soft-mist inhalers, breath-actuated inhalers, and nebulizers. Of these, pressurized metered-dose and soft-mist inhalers are active-spray devices, while dry-powder inhalers are passive-spray devices that depend on the patient’s inspiratory-flow rate (3). Despite their advantages, the maximum therapeutic potential of these various devices has not been realized. Surveys of inhaler use among patients with chronic obstructive pulmonary disease show that medication adherence to various inhalers is remarkably low, only 10%–40% (4, 5), and the accuracy rate of inhalation techniques can be as low as 22.7%–31.8% (6, 7). Non-standardized inhaled therapy often leads to suboptimal treatment outcomes that increase the frequency and severity of acute exacerbations. This aggravates the health and economic burdens of patients, and creates enormous financial waste in healthcare systems (8, 9). Therefore, the standardization of inhaled therapy application is essential.

Inhaled therapy standardization practices rely on professional education and guidance from healthcare professionals, including physicians, pharmacists, and nurses. Both the Global Initiative for Chronic Obstructive Lung Disease (GOLD) and the Global Initiative for Asthma (GINA) guidelines emphasize that clinicians must educate patients regarding the proper method of inhaled therapy. They also suggest the assessment of patients’ inhalation technique proficiencies and reiteration of the instructions during follow-up visits (10, 11). Nurses are often the primary providers of inhaled therapy guidance (ITG) due to their role in delivering therapies and the high frequency of nurse–patient contact, which is indispensable during the inhalation treatment process (12, 13). Existing research confirms that nurse-delivered ITG directly enhances patients’ correct inhaler usage and adherence, relieving them of respiratory symptoms in daily life (14). Reznik et al. (15) found that when relatives and primary caregivers receive nurse-led education on inhalation medication regimens and how to operate an inhaler, their subsequent support and supervision yield greater benefits for patients.

However, other relevant studies have shown that ITG provided by clinical nurses currently remains suboptimal, with inconsistent proficiency levels. Maepa et al. (16) surveyed 133 healthcare professionals from multiple hospitals regarding their metered-dose inhaler (MDI) instructions and found that 40% of providers reported never demonstrating MDI operation steps to patients, while 57% admitted to never assessing patients’ inhalation technique during consultations. Huang et al. (17) demonstrated that only 1.9% of community healthcare providers in China conducted repeat education sessions or proactive follow-up assessments for patients regarding inhaled therapy. An analysis of contributing factors suggested that nurses’ insufficient theoretical knowledge of inhaled therapy undermines their confidence and effectiveness in delivering professional guidance. Research indicates that only 10.4% of primary-care providers can identify three or more types of inhaled medications, with nurses particularly lacking in the operational knowledge of various inhalers (18). As direct role models for patients learning inhaler techniques, a nurses’ own inhalation skills significantly impact their instructional quality. Swami et al.’s (19) evaluation of respiratory-ward nurses’ techniques across different devices revealed an average correct operation rate of just 47%. The chronic nature of respiratory diseases requires long-term standardized inhaled therapy, and respiratory nurses as the primary managers of these patients play a crucial role in ensuring proper inhaler adherence. Consequently, their ability to effectively translate comprehensive theoretical knowledge into practical clinical-guidance skills during patient education is crucial.

However, the current research on nurses’ ITG has primarily focused on their mastery of theoretical knowledge, such as inhalation drugs and inhaler operation techniques, along with rudimentary assessments of the guidance frequency and forms (20–22). Reliance only on existing findings to evaluate respiratory nurses’ ITG competency yields an incomplete perspective. Moreover, most studies have concentrated on tertiary hospitals and survey mixed groups of physicians and nurses. This has resulted in an underexplored issue of systemic competency disparities driven by the hierarchical distribution of healthcare resources (23, 24). In addition, the tools used in recent studies regarding ITG by nurses were primarily designed by the researchers themselves and lacked theoretical foundations or reliability/validity testing. Therefore, their scientific rigor remains questionable, which limits the generalizability of their conclusions. Anderson’s knowledge taxonomy divides knowledge into four domains: (1) factual knowledge (“knowing what”); (2) conceptual knowledge, which encompasses theoretical understanding (“knowing why”); (3) procedural knowledge (“knowing how to do something”); and (4) cognitive knowledge, which is further subdivided into (a) technical knowledge (understanding operational sequences) and (b) practical knowledge (executing actions to teach others) (25). Put in these terms, nurses’ ITG must be systematically transformed from factual to procedural knowledge (26), integrating theoretical comprehension with practical-skill execution to develop comprehensive competency.

In this study, we assess the self-reported ITG competency among respiratory nurses across multiple-tier healthcare institutions and identify influencing factors. Our ultimate goal is to provide evidence for policy-makers to develop stratified interventions and optimize training resource allocation across the healthcare system.

## 2 Methods

### 2.1 Data collection instruments

An ITG competency scale was developed based on Anderson’s knowledge taxonomy to ensure theoretical depth. To ensure content validity, the specific items regarding inhaler operation and guidance were rigorously mapped to the standardized procedural steps outlined in the GOLD and GINA guidelines (10, 11). This ensured that an assessment of “correct technique” was grounded in internationally recognized clinical standards rather than subjective interpretation.

The survey questionnaire consisted of two portions. The first portion collected nurses’ demographic data, such as age, gender, highest educational-attainment level, and marital status. The second portion was comprised of 35 questions related to ITG competency, including two key dimensions: knowledge of ITG and skills related to administering inhaled therapy. The knowledge dimension included 21 items that evaluated the respondents’ levels of knowledge regarding inhalant drugs, the clinical application of inhalers, and inhaler operation. Each question was assigned a value of one (1) point for yes or zero (0) points for no, with higher scores indicating greater knowledge. The skill dimension included 14 items that evaluated nurses’ skills in administering inhaled therapy. This focused on ITG implementation and inhalation technique proficiency and application. These items were rated on a Likert scale (1 = strongly disagree to 5 = strongly agree), with higher scores indicating better inhalant instruction skills.

To validate the questionnaire, two subsequent rounds of expert consultation were conducted using the Delphi method, and 10 experts were invited to evaluate the scale. The expert authority coefficient for each round was 0.840. The validity and reliability of the instrument were subsequently examined.

### 2.2 Study design and population

A cross-sectional study based on a questionnaire survey was conducted from March 2022 to February 2023 in multilevel hospitals in Jiangsu Province, Eastern China. In accordance with the *2020 China Health Statistics Yearbook*, which provides data on the distribution of public multilevel hospitals in Jiangsu Province, respiratory nurses were selected from 5 tertiary, 5 secondary, and 10 primary hospitals using cluster sampling.

The sample size was calculated based on the following equation (27):

$$n={\left(\frac{Z_{1-\alpha /2}\sigma }{\delta }\right)}^{2}$$

where *α* = 0.05, *Z*_1 − α/2_ = 1.96, and *δ* represent the allowable error that was set to not exceed 0.1. According to a previous study (18), the average ITG score of respiratory nurses was 2.70 ± 1.26 points. Hence, the standard deviation (SD) *σ* was determined to be 1.26. Assuming a 10% invalid response rate, we estimated that a sample size of at least 671 participants was required. The nurses used the questionnaire to self-rate their performances.

All participants met the following inclusion criteria: (a) on-the-job nurses who had obtained a nurse practicing certificate; (b) previous engagement in either: (i) respiratory nursing in respiratory wards (regardless of hospital level) for a continuous period of ≥1 year, or (ii) inhaled therapy work (i.e., engaged in teaching inhaler technique) at a primary hospital for a continuous period of ≥1 year; and (c) informed consent/voluntary participation in this study. Exclusion criteria were nurses who (a) were undergoing further education, those in probation, and interns; or (b) were not on duty during the survey period. All eligible nurses completed the survey through Wenjuanxing, an electronic survey platform in China. Written informed consent was obtained through the electronic consent embedded in the survey platform. Participants were required to read the consent information and agree prior to continuing the survey. Each device could only submit one response, and IP address verification was used to prevent duplicate answers.

### 2.3 Scoring logic

The ITG competency scale consisted of two dimensions: knowledge of ITG and skills related to inhaled therapy administration, with a total of 35 items. The knowledge dimension consisted of 21 items that assessed the participants’ knowledge of inhalant drugs, the clinical application of inhalers, and inhaler operation. Items were binary scored (correct = 1, incorrect = 0), yielding a raw knowledge score ranging from 0 to 21. The skills dimension consisted of 14 items that assessed ITG implementation and inhalation technique application. Items were rated on a 5-point Likert scale ranging from 1 (strongly disagree) to 5 (strongly agree), yielding a raw skills score ranging from 14 to 70. To facilitate comparison across dimensions, the raw scores were transformed to a 100-point scale using the following equation:

$$\text{Standardized score}=(\begin{array}{l} \mathrm{raw}\;\text{score}/\text{maximum}\;\\ \text{possible}\;\mathrm{raw}\;\text{score} \end{array})\times 100$$

Based on the standardized total competency score, participants were classified into three categories: poor (<60 points), medium (60–85 points), and good (>85 points). These categories were determined according to predefined criteria used in previous studies (28).

### 2.4 Data analysis

Statistical analysis was performed using SPSS v22.0 (IBM Corp., Armonk, NY, USA). Statistical significance was set at *p* < 0.05. Categorical data were described as the number, frequency, and percentage of participants, while continuous data were described as means ± SDs. A univariate analysis was performed using an independent-sample *t* test and one-way analysis of variance (ANOVA). Chi-square testing with 3 × 3 contingency tables was initially conducted to assess the distributional differences in competency performance tiers (poor, medium, and good) across multi-tier hospitals. After significant heterogeneity in competency distribution was identified, *post hoc* Bonferroni-adjusted pairwise comparisons were performed between hospital tiers. Pearson’s correlation coefficient was used to examine relationships among knowledge, skills, and competency. A hierarchical regression analysis was performed with ITG competency for respiratory nurses as the dependent variable. Variables significantly associated with ITG competency in the univariate analysis were entered into the model. In the first block, the hospital level was included as a dummy variable (with primary hospitals serving as the reference) to control for potential systemic differences in clinical competency across hospitals. In the second block, these variables (which were significantly correlated with ITG competency) were added to assess their incremental contribution. The changes in the standardized coefficient beta (*β*) and R^2^ were used to evaluate the relative association between the variables and ITG competency. A higher standardized coefficient beta (*β*) value and R^2^ change indicated a greater relative association. Multicollinearity was assessed using the variance inflation factor (VIF) and tolerance levels. In this study, the VIF values ranged from 1.061 to 6.086, and the tolerance ranged from 0.164 to 0.943, indicating no significant multicollinearity among the independent variables.

### 2.5 Ethical considerations

This study was approved by the Ethics Committee of the First Affiliated Hospital of Soochow University (Suzhou, China; SDFY2022 No.462). The study was conducted in accordance with the Declaration of Helsinki. Informed consent was obtained from all participants. Participation was voluntary, and informed consent was obtained through the Wenjuanxing online survey platform. No personal identification information was collected, and the confidentiality and anonymity of all respondents was ensured.

## 3 Results

The self-reported questionnaire demonstrated satisfactory reliability. Cronbach’s *α* coefficient was 0.769 for the knowledge dimension, 0.964 for the skills dimension, and 0.903 for the entire questionnaire. The questionnaire’s scale-level content validity index was 0.857. An exploratory-factor analysis yielded a Kaiser–Meyer–Olkin value of 0.917, indicating an adequate sample adequacy for the factor analysis. The result of a Bartlett’s test of sphericity was significant (*p* < 0.001), indicating that the questionnaire was suitable for a further exploratory-factor analysis. Based on scree plots analysis, eigenvalues plateaued after the second or third factor, and the factor structure aligned with the two theoretically proposed dimensions: knowledge of ITG and skills related to inhaled therapy administration.

Of the 999 nurses we recruited, 983 met the inclusion criteria. Twenty-one were excluded due to incomplete data, leaving a total of 962 nurses who were enrolled in the study. This study involved a total of 962 respiratory nurses working at multilevel hospitals, of whom 151 (15.70%) worked at primary hospitals, 309 (32.12%) at secondary hospitals, and 502 (52.18%) at tertiary hospitals. Of all the participants, 86.80% acquired ITG knowledge through department-organized training and lectures, 80.87% via the instruction sheets provided with medications, 74.22% by accessing video and text resources from the internet, and 73.60% relied on clinical-work experience. Regarding the distribution of ITG training methodologies, 73.60% of nurses received training in specialized lectures, 68.61% underwent hands-on training, 56.13% participated in online training programs, and 53.85% engaged in simulation-based training. Moreover, statistically significant differences in ITG competency were observed, which depended on varying sources of inhaled therapy knowledge and different training methodologies (*p* < 0.001). ITG competency differences organized by the general characteristics of multilevel hospitals are shown in Table 1.

**Table 1:** Differences in inhaled therapy guidance competency by the general characteristics of multilevel hospitals.

Variable	Primary hospitals (*n* = 151)			Secondary hospitals (*n* = 309)			Tertiary hospitals (*n* = 502)			Overall (*n* = 962)		
	Frequency (%)	Score	Test results	Frequency (%)	Score	Test results	Frequency (%)	Score	Test results	Frequency (%)	Score	Test results
Gender			−0.699			—			−2.101			−2.227
Male	4 (2.60)	66.50 ± 8.35	0.486	0	—	—	11 (2.20)	69.27 ± 8.95	0.036	15 (1.56)	68.53 ± 8.58	0.026
Female	147 (97.40)	70.69 ± 11.91		309 (100)	74.12 ± 8.75		491 (97.8)	74.88 ± 8.75		947 (98.44)	73.98 ± 9.41	
Age (y)			0.038			1.241			14.463			4.110
≤25	31 (20.50)	70.58 ± 10.34	0.990	78 (25.20)	74.46 ± 9.08	0.295	99 (19.70)	71.05 ± 9.38	0.000	208 (21.62)	72.26 ± 9.52	0.007
26–35	76 (50.30)	70.68 ± 12.36		162 (52.40)	74.70 ± 8.55		290 (57.80)	74.70 ± 8.75		528 (54.89)	74.12 ± 9.38	
36–45	39 (25.80)	70.21 ± 12.53		67 (21.70)	72.42 ± 8.87		95 (18.90)	77.71 ± 6.80		201 (20.89)	74.49 ± 9.35	
≥45	5 (3.30)	72.00 ± 8.92		2 (0.60)	70.50 ± 3.54		18 (3.60)	80.56 ± 6.85		25 (2.60)	78.04 ± 8.00	
Highest educational- attainment level			4.806			1.497			4.315			8.409
Junior college	52	67.98 ± 12.77	0.009	76	74.04 ± 8.72	0.225	91	72.20 ± 9.94	0.021	219 (22.77)	71.84 ± 10.51	0.000
Undergraduate degree	65	69.96 ± 11.74		201	73.75 ± 8.90		395	75.23 ± 8.38		661 (68.71)	74.26 ± 9.04	
Master’s degree or above	34	75.74 ± 8.78		32	76.63 ± 7.68		16	77.69 ± 9.27		82 (8.52)	76.46 ± 8.39	
Marital status			0.624			0.233			8.317			4.283
Unmarried	53	71.49 ± 10.00	0.537	90	73.71 ± 9.75	0.792	155	72.29 ± 9.27	0.003	298 (30.98)	72.58 ± 9.55	0.014
Married	94	70.31 ± 12.74		213	74.24 ± 8.33		340	75.82 ± 8.34		647 (67.25)	74.50 ± 9.28	
Divorced or other	4	65.00 ± 12.83		6	75.83 ± 8.75		7	78.00 ± 8.49		17 (1.77)	74.18 ± 10.56	
Professional title			0.314			0.525			8.783			4.101
Nurse	41	70.98 ± 10.00	0.816	69	73.77 ± 9.28	0.665	87	71.44 ± 9.83	0.000	197 (20.48)	72.16 ± 9.70	0.007
Senior nurse	46	69.26 ± 12.72		129	74.37 ± 8.86		205	74.42 ± 8.54		380 (39.50)	73.78 ± 9.37	
Supervisor nurse	51	71.53 ± 13.16		95	74.44 ± 8.21		159	75.80 ± 8.38		305 (31.70)	74.66 ± 9.39	
Co-chief superintendent nurse or above	13	70.31 ± 8.71		16	71.69 ± 9.02		51	78.55 ± 7.09		80 (8.32)	75.84 ± 8.49	
Work experience (y)			0.152			0.491			9.663			2.820
1–5	52	69.92 ± 11.64	0.928	110	74.87 ± 8.79	0.689	167	72.50 ± 9.36	0.000	329 (34.20)	72.88 ± 9.69	0.038
6–10	33	71.09 ± 11.14		97	74.00 ± 8.64		149	74.70 ± 8.45		279 (29.00)	74.03 ± 8.91	
11–20	52	71.19 ± 13.25		90	73.42 ± 8.76		145	76.19 ± 8.19		287 (29.84)	74.42 ± 9.63	
≥21	14	69.57 ± 9.08		12	73.42 ± 9.89		41	79.12 ± 7.04		67 (6.96)	76.10 ± 8.87	
Years of specialization			2.267			2.196			9.371			0.766
1–5	101	72.03 ± 10.68	0.083	206	74.76 ± 8.59	0.089	246	73.12 ± 9.40	0.000	553 (57.48)	73.53 ± 9.40	0.513
6–10	30	69.50 ± 14.25		76	73.25 ± 8.77		170	75.50 ± 8.23		276 (28.69)	74.23 ± 9.36	
11–20	16	65.56 ± 12.80		26	72.19 ± 9.42		71	77.59 ± 6.85		113 (11.75)	74.65 ± 9.48	
≥21	4	62.25 ± 9.95		1	—		15	79.87 ± 6.49		20 (2.08)	75.25 ± 10.71	
Employment			0.195			0.928			4.523			3.076
Officially on staff	57	70.82 ± 11.47	0.846	120	74.70 ± 7.91	0.354	142	77.33 ± 7.56	0.000	319 (22.16)	75.18 ± 8.80	0.002
Contract	94	70.44 ± 12.10		189	73.75 ± 9.25		360	73.74 ± 9.04		643 (66.84)	73.26 ± 9.66	
Have you administered inhaled therapy?			3.130			3.659			0.012			3.304
Yes	33	74.94 ± 7.82	0.002	89	76.64 ± 7.02	0.000	112	74.77 ± 8.96	0.990	234 (24.3)	75.50 ± 8.12	0.001
No	118	69.36 ± 12.48		220	73.10 ± 9.18		390	74.76 ± 8.75		728 (75.7)	73.38 ± 9.75	
Have you been responsible for inhaled therapy training?^#^			2.585			0.530			4.476			5.390
Yes	21	75.24 ± 8.25	0.014	104	74.49 ± 8.51	0.597	234	76.59 ± 8.13	0.000	359 (37.3)	75.90 ± 8.28	0.000
No	130	69.83 ± 12.16		205	73.93 ± 8.89		268	73.16 ± 9.04		603 (62.7)	72.70 ± 9.85	
Have you received inhaled therapy training?^$^			3.399			0.981			3.984			5.636
Yes	83	73.54 ± 9.28	0.001	251	74.35 ± 8.70	0.327	455	75.36 ± 8.35	0.000	789 (82.0)	74.85 ± 8.58	0.000
No	68	66.97 ± 13.55		58	73.10 ± 8.99		47	68.96 ± 10.68		173 (18.0)	69.57 ± 11.65	

# Refers to whether a nurse is responsible for delivering inhaled therapy training to patients or other healthcare staff within their department.

$ Refers to whether a nurse has received formal training in ITG such as specialized lectures, hands-on workshops, or simulation-based programs.

**Figure 1:**
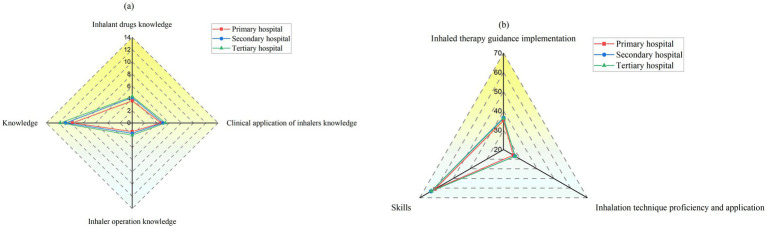
Radar graphs of the knowledge and skill dimensions in multilevel hospitals. **(a)** The knowledge dimension and its three subdomains: knowledge on inhalant drugs, clinical application of inhalers, and inhaler operation. **(b)** The skill dimension and its two subdomains: ITG implementation, and inhalation technique proficiency and application.

The average ITG competency score was 73.90 ± 9.42, with knowledge and skill dimensions scoring 11.14 ± 3.37 and 62.76 ± 8.53, respectively. The knowledge dimension consisted of three subdomains: pharmacological knowledge of inhaled medications (4.16 ± 1.57), clinical application of inhalers (5.16 ± 1.81), and operational knowledge of inhalers (1.81 ± 1.06). The skill dimension consisted of guidance implementation (36.04 ± 4.79) and inhalation technique proficiency and application (26.72 ± 4.01). The interhospital tier comparisons demonstrated statistically significant differences in all competency domains (*p* < 0.05) except for application of guidance skills, where the disparity approached significance (*p* = 0.052). Radar charts for the multilevel hospitals are shown in Figure 1.

**Figure 2:**
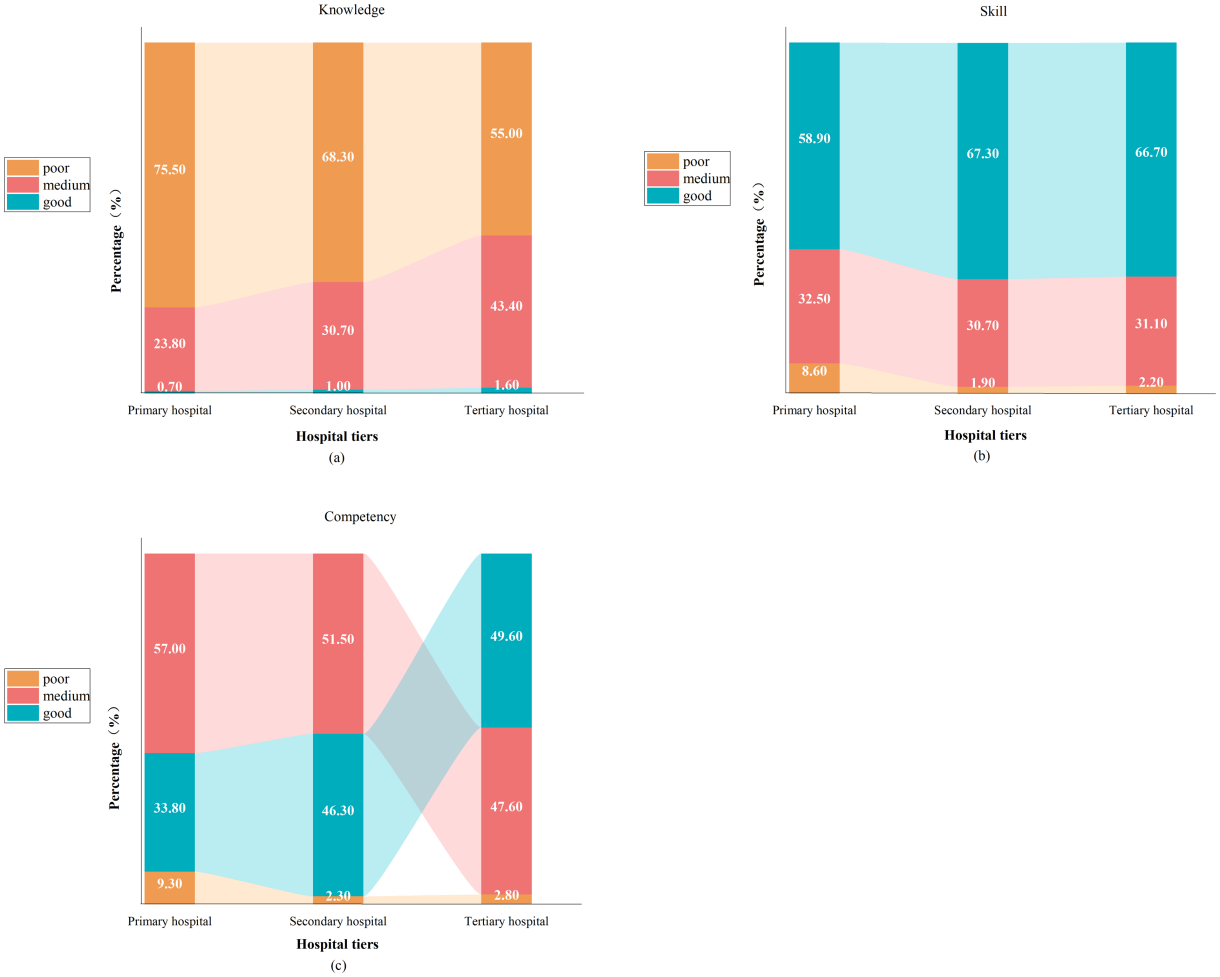
Proportions of ITG competency levels across hospital grades and performance tiers. Stacked-bar plot depicting proportions of knowledge **(a)**, skills **(b)**, and competency **(c)** in ITG, stratified by hospital tier and standardized performance level (poor: <60 points, medium: 60–85 points, good: >85 points).

The standardized competency scores and performance level classifications (Scoring logic in Methods) revealed significant disparities in ITG competency across hospital tiers (*χ*^2^ = 24.219, *p* < 0.001), with primary hospitals demonstrating higher deficiency rates and lower proportions of proficient nurses than secondary and tertiary hospitals (all *p* < 0.001). Tertiary hospitals had minimal knowledge deficiencies, while skill-related deficiencies were disproportionately concentrated in primary-care facilities despite comparable skill levels across all tiers (all *p* < 0.001). The specific patterns are illustrated in Figure 2 below.

A correlation analysis was conducted to examine the relationship between knowledge and skills as two independent dimensions. The results showed a significant positive correlation between knowledge and skills (*r* = 0.082, *p* < 0.05).

A multivariate linear-regression analysis was used to predict the factors that influence respiratory nurse ITG competency. The results showed that the highest level of educational attainment, hospital grade, and training methodologies significantly affected competency (Table 2). Respiratory nurses with master’s degrees or above, those working in tertiary hospitals, and/or those trained via specialized lectures or simulation-based training had greater ITG competency.

**Table 2:** Hierarchical regression analysis of the correlation factors of ITG competency.

Model	Independent variables	*β*	*SE*	*t*	*p* value
Model 1	Hospital grade				
	Secondary hospital	3.537	0.925	3.823	0.000
	Tertiary hospital	4.176	0.865	4.829	0.000
Model 2	Hospital grade				
	Tertiary hospital	2.205	0.949	2.324	0.020
	Highest educational-attainment level				
	Master degree or above	4.864	1.368	3.556	0.000
	Training methodologies				
	Specialized lectures	2.073	0.917	2.260	0.024
	Simulation-based training	2.218	0.769	2.885	0.004

SE, standard error. Model 1: *R*^2^ = 0.024, F = 11.791, *p* < 0.001; Model 2: *R*^2^ = 0.140, Δ*R*^2^ = 0.118, F = 5.748, *p* < 0.001.

## 4 Discussion

Inhaled therapy is an important administration route for patients with chronic airway diseases, and respiratory nurses’ competency in delivering ITG plays a key role in ensuring standardized medication use among patients (29, 30). In this study, a self-reported competency scale for ITG respiratory nurses was developed and validated, and it demonstrated satisfactory reliability and validity. The primary results indicated that respiratory nurses exhibited moderate levels of ITG competency, and they scored higher in skills than in knowledge, particularly in primary hospitals. A hierarchical regression analysis identified higher educational attainment, hospital grade, and training through specialized lectures or simulations as significant factors associated with higher ITG competency scores.

Our results showed that respiratory nurses generally exhibited inadequate proficiency in the provision of guidance of inhalation therapy, with less than 50% of them attaining a “good” rating (Figure 2c). Significant demographic disparities in ITG competency across multilevel hospitals in Eastern China were found, with tertiary hospitals exhibiting broader demographic influences than primary institutions. In primary hospitals, competency was narrowly associated with basic education and training-related factors such as prior inhalation experience, training responsibility (i.e., whether nurses were responsible for delivering inhaled therapy training), and training participation (i.e., whether nurses had received formal training in inhaled therapy). The univariate analyses results showed that training participation was significantly associated with ITG competency in all hospital tiers except at secondary hospitals, where prior inhalation experience showed a significant association. This results agreed with the evidence showing that targeted training bridges knowledge–practice gaps for nurses (31). However, hierarchical disparities persisted, with tertiary hospitals demonstrating substantially greater access to systematic training programs than primary institutions, where training opportunities remained fragmented and inconsistent. Additionally, the competency gap reflected disparities in resource allocation. Rural and community hospitals often lacked structured training programs, relying on *ad hoc* skill transfer. For example, 67.5% of primary nurses received oral instructions to fulfill their training requirements, whereas nurses in tertiary hospitals benefited from diverse training approaches, that included simulation exercises and hands-on practice. Our results revealed that tertiary centers possessed a higher concentration of expertise (e.g., undergraduate degree or head nurses: 81.87% in tertiary *vs.* 65.56% in primary), while primary institutions struggled with fragmented training access (32). These results underscore the urgency of tier-specific training frameworks to address competency gaps, particularly for frontline nurses in resource-constrained settings.

In accordance with Anderson’s knowledge taxonomy, ITG competency for respiratory nurses includes two key dimensions: knowledge of ITG, and skills related to administering inhaled therapy (consistent with the scree plots). Our results showed that ITG knowledge scores across hospitals of various grades were all relatively low, with the highest proportion of knowledge gaps found in primary hospitals (75.50%, 68.30%, 55.00%) (Figure 2a). This results agrees with that of Karle et al. and further validates the current landscape of suboptimal inhalation techniques and heterogeneous inhalation compliance among patients with chronic airway disease (33, 34). This phenomenon might be primarily attributable to the heavy clinical workloads of nurses, which can lead them to prioritize standardized and task-oriented, routine tasks over acquisition of ITG knowledge, thereby creating a knowledge–practice gap in this specialized domain. Additionally, a wide variety of inhalation devices have rapidly come onto the market and nurses often struggle to accurately differentiate between inhaler types and update their knowledge of them in a timely manner. Therefore, we recommend that, while emphasizing the importance of ITG to nurses, managers should also develop targeted training programs focused on ITG and schedule additional learning activities regarding updates to inhalers.

A further analysis revealed that within the knowledge dimension (Figure 1a), nurses scored highest in the clinical application of inhalers, followed by pharmacological knowledge and operational knowledge of inhalers. Nurses at primary hospitals generally scored lower across all subdomains and significantly lagged behind those at tertiary hospitals, particularly in clinical application. This phenomenon might be attributable to the distinctive attributes of this subdomain, the core aspects of which (e.g., patient preference assessment, device replacement judgment, personalized selection) essentially belong to the category of clinical decision making (35). This disparity may be due to an imbalance in resource allocation within China’s tiered healthcare system. The limited supply of inhaled medications and simpler clinical scenarios in primary care restrict opportunities for practice (36). Unlike in tertiary hospitals, which benefit from comprehensive formularies and a concentration of complex cases via the tiered referral system (37), primary hospitals offer fewer opportunities for nurses to engage in advanced decision-making, such as device switching. Compounding this limited clinical exposure is the generalist nature of primary care nursing. These nurses must manage a broad spectrum of clinical areas (38), and they often prioritize immediate clinical demands. This inevitably limits opportunities for the sustained reflective practice required for ITG.

The present study revealed an imbalance in the knowledge-skills of respiratory nurses’ ITG competency, with the self-rating score of the skill dimension significantly exceeding that of the knowledge dimension (Figure 2). This result is contrary to the expectations of Anderson’s theory of knowledge classification, which states that a solid knowledge base is a prerequisite for skill proficiency. In our study, however, a self-reported skill was better than a knowledge score, suggesting that there may be desynchronized development between the two. The rationale that underpins this desynchronization pertains to the prevailing orientation within the contemporary domestic nurse training system that places greater emphasis upon operative rather than mechanistic understanding. Nurses in Chinese hospitals face substantial clinical pressure due to high patient-to-nurse ratios (e.g., 1:12.1 in tertiary and up to 1:15 in primary institutions) (39). According to the cognitive load theory, when clinical demands exceed an individual’s cognitive capacity, learners tend to prioritize procedural memory for immediate problem-solving while neglecting declarative knowledge that requires deeper processing (40). This phenomenon may, at least in part, elucidate the propensity of nurses to cultivate operational competencies within the circumscribed allocation of educational resources. Current on-the-job training predominantly employs the one-way indoctrination model of “demonstration-imitation.” This approach emphasize the standardization of operational steps but often overlooks the underlying principles of clinical decision-making. For example, training focused on inhaler device assembly and utilization may provide limited guidance on the rationale for the selection of specific inhaler types (e.g., dry powder vs. soft mist) based on patient needs. While enhancing short-term operational proficiency, this paradigm hinders the development of higher-order decision-making skills. Consequently, clinical experience remains “tacit” and is not systematically integrated into a declarative knowledge system. This leads to a gap between procedural skills and conceptual understanding.

The results of this study showed that ITG implementation scores were higher than those for inhalation technique proficiency (Figure 1b). Notably, significant differences between hospital tiers were observed only in implementation, while technique proficiency and application showed a similar trend across all hospital levels. This may be because implementation relies heavily on soft skills, such as patient communication and proactive learning, which depend more on individual initiative than on hospital resources. With the widespread adoption of digital platforms and the growing emphasis on patient-centered care (41–43), the gap in soft skill resources between hospitals of different levels is narrowing (44, 45).

In this study, nurses with higher educational-attainment levels achieved higher competency scores than did other nurses. This might be because their advanced education cultivated stronger self-directed learning, heightened self-expectations, and greater intrinsic motivation, driving systematic improvements in both theoretical knowledge and practical skills (46, 47). Furthermore, the superior performances of nurses in higher-tier hospitals agreed with the uneven distribution of medical resources in China, where high-quality resources and systematic training opportunities are concentrated in tertiary centers, leading to enhanced nursing proficiency. This enables the nurses in tertiary centers to access systematic training and assessments, ultimately enhancing their ITG competency (48, 49). This result agreed with the viewpoint of Wang Fen et al. (50). It is also worth mentioning that our results showed that specialized lectures and simulation training were particularly effective for enhancing ITG competency in respiratory nurses. Unlike textbooks, which often lag behind in regard to clinical advances, lectures deliver up-to-date knowledge on new techniques, building a strong foundation for practice. Additionally, simulation training immerses nurses in real-world scenarios (51). By managing exacerbations of chronic obstructive pulmonary disease and guiding elderly patients through inhaler techniques, nurses repeatedly practice and refine both knowledge application and procedural skills to achieve operational automaticity and proficiency. It is recommended that a stratified training framework be tailored to hospital tiers. For primary hospitals, interventions could focus on vertical resource transfer through the establishment of regional “respiratory nursing alliances” to enable tertiary centers to provide digital resources and mentorship focused on essential skills such as device identification and standardized operational techniques. Conversely, training in tertiary hospitals is encouraged to emphasize clinical reasoning and the utilization of high-fidelity simulations (e.g., acute exacerbations) to refine complex decision-making. Furthermore, given that a simulation is a strong predictor of competency, low-fidelity role playing is suggested as a cost-effective alternative in resource-limited settings to universally bridge the theory practice gap.

This study has several limitations. First, due to the absence of individual hospital identifiers in the original dataset, we were unable to account for the clustering of nurses within hospitals using multilevel analysis. We did include the hospital level (primary, secondary, or tertiary) as a dummy variable in the hierarchical regression analysis to partially adjust for institutional differences, but future studies should consider analyzing the nested structure of the data. Second, the primary results were based on participants’ self-reported competency. Although the self-rating tool demonstrated good reliability, such measures are inherently susceptible to social desirability bias, and participants may have overestimated or underestimated their own competency. Consequently, the results might not fully reflect their actual ITG competency. Third, the investigation was exclusively conducted in Jiangsu Province, Eastern China. Thus, our results may lack generalizability to other areas of China, particularly in western regions with fewer training resources. Finally, because the total competency score was mathematically derived from the knowledge and skills dimensions, correlations involving the total score are at least partially subject to part-whole correlations. Therefore, our analysis focused only on the relationship between the two independent subdimensions, which may not have captured a holistic understanding of how knowledge and skills jointly contribute to overall competency.

## 5 Conclusion

This study highlighted the importance of ITG competency among respiratory nurses for the standardization of inhaled therapy. While the overall skills related to administering inhaled therapy were satisfactory, notable deficits in theoretical knowledge were observed, particularly among nurses in primary hospitals. These results suggest a potential imbalance in the distribution of educational resources across hospital tiers. In addition, higher educational attainment, hospital grade, and training via specialized lectures or simulations were found to be associated with greater competency. Based on these observations, future efforts should consider the optimization of resource allocations through stratified interventions to facilitate vertical resource transfer from tertiary centers to primary care. Additionally, standardized, high-quality training programs should be promoted to support competency homogenization across all hospital tiers. Future research using longitudinal or interventional designs is required to test the effectiveness of these approaches.

## Data Availability

The datasets presented in this article are not readily available because the data are available only on request and are not publicly available due to privacy or ethical restrictions. Requests to access the datasets should be directed to Hxkzhaoxi@163.com.
